# Immune cell census in murine atherosclerosis: cytometry by time of flight illuminates vascular myeloid cell diversity

**DOI:** 10.1093/cvr/cvy109

**Published:** 2018-05-02

**Authors:** Jennifer E Cole, Inhye Park, David J Ahern, Christina Kassiteridi, Dina Danso Abeam, Michael E Goddard, Patricia Green, Pasquale Maffia, Claudia Monaco

**Affiliations:** 1 Kennedy Institute of Rheumatology, Nuffield Department of Orthopaedics, Rheumatology and Musculoskeletal Sciences, University of Oxford, Oxford OX3 7FY, UK; 2 Centre for Immunobiology, Institute of Infection, Immunity and Inflammation, College of Medical, Veterinary and Life Sciences; 3 Institute of Cardiovascular and Medical Sciences, College of Medical, Veterinary and Life Sciences, University of Glasgow, Glasgow G12 8TA, UK; 4 Department of Pharmacy, University of Naples Federico II, 80131 Naples, Italy

**Keywords:** Immune cells, Macrophages, Atherosclerosis, Vascular, Cytometry by time of flight

## Abstract

**Aims:**

Atherosclerosis is characterized by the abundant infiltration of myeloid cells starting at early stages of disease. Myeloid cells are key players in vascular immunity during atherogenesis. However, the subsets of vascular myeloid cells have eluded resolution due to shared marker expression and atypical heterogeneity in vascular tissues. We applied the high-dimensionality of mass cytometry to the study of myeloid cell subsets in atherosclerosis.

**Methods and results:**

Apolipoprotein E-deficient (ApoE^−/−^) mice were fed a chow or a high fat (western) diet for 12 weeks. Single-cell aortic preparations were probed with a panel of 35 metal-conjugated antibodies using cytometry by time of flight (CyTOF). Clustering of marker expression on live CD45^+^ cells from the aortas of ApoE^−/−^ mice identified 13 broad populations of leucocytes. Monocyte, macrophage, type 1 and type 2 conventional dendritic cell (cDC1 and cDC2), plasmacytoid dendritic cell (pDC), neutrophil, eosinophil, B cell, CD4^+^ and CD8^+^ T cell, γδ T cell, natural killer (NK) cell, and innate lymphoid cell (ILC) populations accounted for approximately 95% of the live CD45^+^ aortic cells. Automated clustering algorithms applied to the Lin-CD11b^lo-hi^ cells revealed 20 clusters of myeloid cells. Comparison between chow and high fat fed animals revealed increases in monocytes (both Ly6C^+^ and Ly6C^−^), pDC, and a CD11c^+^ macrophage subset with high fat feeding. Concomitantly, the proportions of CD206^+^ CD169^+^ subsets of macrophages were significantly reduced as were cDC2.

**Conclusions:**

A CyTOF-based comprehensive mapping of the immune cell subsets within atherosclerotic aortas from ApoE^−/−^ mice offers tools for myeloid cell discrimination within the vascular compartment and it reveals that high fat feeding skews the myeloid cell repertoire toward inflammatory monocyte-macrophage populations rather than resident macrophage phenotypes and cDC2 during atherogenesis.

## Introduction

Inflammation underlies the pathogenesis of atherosclerosis, the most common cause of cardiovascular disease (CVD)^1^. The Canakinumab Anti-inflammatory Thrombosis Outcomes Study (CANTOS) trial recently offered proof that targeting inflammation could be beneficial in CVD^2^. Mapping immune cell subsets and their activation in CVD will allow discrimination between pathogenic and reparative subtypes, thus aiding selective diagnostic and therapeutic approaches and uncoupling of homeostatic and tissue damaging cell function.

Development of atherosclerosis is defined by the presence of mononuclear phagocytes in the subendothelial space. Monocytes in the circulation adhere to the activated endothelium of arteries and migrate into the subendothelial space where they differentiate into macrophages under the effects of growth factors. Previously, it was thought that macrophage populations could be simplistically divided into subsets such as M1 and M2 akin to T lymphocyte populations. However, growing evidence supports a complex continuum of macrophage activation states, especially in diseased tissue where dynamic environmental cues exist^3^. Myeloid cells, particularly macrophages, play critical roles in the progression and regression of atherosclerosis including lipid uptake, antigen presentation, dead cell removal and cytokine production and atherosclerotic mice lacking macrophages are protected from disease development^4–6^. Tissue resident macrophage populations are also present in the vessel wall^7^.

To date, studies have relied on immunohistochemistry, gene expression assays and flow cytometry to identify these populations in cardiovascular tissues. Whilst these techniques have taught us a great deal, they also have limitations. Delineation of different myeloid cell populations requires multi-parameter analysis as expression of many antigens is overlapping between several cell types, e.g. CD11c is expressed by both dendritic cells and macrophages^8^. In addition, multiple markers are required to facilitate definition of various subsets of the same cell-type, e.g. type 1 and type 2 conventional dendritic cells. This is challenging in tissues such as murine aortas where cell numbers are small and autofluorescence is a severe limitation. Thus, despite the identification of several macrophage markers in atherosclerosis, a full description of the vascular macrophage populations has been hampered by a lack of available technology.

Cytometry by time of flight (CyTOF) combines flow cytometry and inductively coupled plasma (ICP) mass spectrometry, the most quantitative and sensitive method of determining the elemental composition of materials^9^. In mass cytometry, stable isotopes of non-biological rare earth metals are used as reporters conjugated to the same clones of antibody that are used in conventional flow cytometry. A major benefit thus being the avoidance of interference by autofluorescence and spectral overlap^10^. CyTOF represents a step change in our ability to phenotype cells in tissues in a broad and high-dimensional manner.

Here, we describe the use of mass cytometry to define the immune cell composition of murine aortas in mild and more advanced atherosclerosis. High fat feeding was found to alter the proportions—rather than the absolute representation—of these subsets, increasing those with an inflammatory myeloid cell phenotype at the expense of those with a resident myeloid cell phenotype and type 2 conventional dendritic cells.

## Methods

### Mice

Apolipoprotein E deficient (ApoE^−^^/^^−^) mice on a C57BL/6 background were purchased from Charles River Laboratories and bred in-house. Mice were weaned at 4 weeks of age. At 12 weeks of age, some mice had the normal chow diet replaced with a cholate-free high fat diet from Special Diets Services (Essex, UK) consisting of (w/w) cocoa butter (15%), cholesterol (0.25%), maize starch (10%), casein (20%), sucrose (40.5%), cellulose (5.95%), corn oil (1%), 50% choline chloride (2%), methionine (0.2%), and mineral mixture (5.1%). Animals were housed under specific-pathogen free conditions and studied according to UK Home Office regulations and institutional guidelines. UK Home office regulations conform to the guidelines from Directive 2010/63/EU of the European Parliament on the protection of animals used for scientific purposes.

### Isolation and digestion of aortas

At 24 weeks of age ApoE^−^^/^^−^ mice were euthanized with an intraperitoneal injection of 250 mg/kg pentobarbital. Tissues were well perfused *in situ* with 20 ml saline via a cannula inserted into the left ventricle (outflow via an incision in the right atrium) to minimize blood cell contamination^11^. Aortas, including the aortic arch, thoracic and abdominal portions were harvested, chopped in to small pieces and incubated for 50 min at 37°C with an enzyme cocktail formulated as previously described^12^. Post-digestion, cells were washed and single-cell suspensions obtained by mashing aortas through a 70 μm cell strainer (Greiner Bio-One).

### Mass cytometry

All directly conjugated antibodies were purchased from Fluidigm and purified unlabelled antibodies from the vendors shown in see Supplementary material online, *Table S1*. Conjugation of purified unlabelled antibodies was performed in house using the Maxpar X8 Metal Labelling Kit (Fluidigm) according to manufacturer’s instructions. All antibodies were titrated to determine the optimal staining concentration.

Due to the low number of cells that are obtained from a single mouse aorta, single cells from two aortas were combined to make one sample. Samples were first stained with rhodium DNA intercalator (Fluidigm) to distinguish live/dead followed by barcoding using the palladium-based 20-Plex Pd Barcoding Kit (Fluidigm) according to the manufacturer’s instructions. Barcoded cells were then combined into a single tube prior to Fc receptor blocking (BD Biosciences) and staining with a mixture of metal-conjugated antibodies (see Supplementary material online, *Table S1*) recognizing cell-surface antigens for 30 min at 4°C. The cells were then washed and permeabilized using the Foxp3/Transcription Factor Staining Buffer Set (eBiosciences) according to manufacturer’s instructions, prior to staining with antibodies against intracellular markers for 30 min at 4°C. Finally, stained cells were incubated with Iridium DNA intercalator (Fluidigm) in Maxpar fix and perm buffer (Fluidigm) for up to 48 h at 4°C. Prior to acquisition, cells were washed with cell staining buffer (Fluidigm) twice followed by two washes with water (Fluidigm) and filtered through 40 um cell strainer before being acquired on a Helios mass cytometer (Fluidigm).

### CyTOF data processing and analysis

Data were in the .fcs file format. All .fcs files in the experiment were normalized and then debarcoded using tools within the Helios software. Normalized and debarcoded .fcs files were then uploaded to Cytobank (www.cytobank.org) for all gating and further analysis using the automated dimensionality reduction algorithm viSNE^13^. For analysis using Phenograph^14^, gated .fcs files were exported from Cytobank into R and analysed using the Bioconductor package Cytofkit (v3.6)^15^. Markers used as the clustering channels for each viSNE and Phenograph analysis are outlined in see Supplementary material online, *Table S1*.

The gating strategy employed in Cytobank is presented in see Supplementary material online, *Figure S1A* and consisted of sequential gating for intact single cells using the iridium DNA intercalator, removal of the normalization beads using a standalone bead channel and gating for cell viability using the rhodium DNA intercalator. CD45^+^ cells were gated based on expression of CD45. Among the CD45^+^ cells, we observed a population of CD4^+^CD8^+^ double positive cells. We hypothesize that these cells are contaminating thymic t-cells as the murine thymus is located in close relation to the aortic arch and it is difficult to dissect the aorta without disturbing the thymus^16^. These double positive cells were excluded from further analyses. For myeloid cell viSNE and Phenograph analysis, cells were gated as Live CD45^+^Lin^-^CD11b^lo-hi^. For T cell viSNE analysis, cells were gated as live CD45^+^CD90.2^+^CD3^+^ and for B cell viSNE analysis cells were gated as Live CD45^+^CD19^+^

### Statistics

Data were analysed with GraphPad Prism (version 7.0a, La Jolla, USA). All data are expressed as Mean ± SD unless otherwise stated. Where data did not pass a normality test, Mann–Whitney *U* tests were performed. An alpha level of .05 was considered as statistically significant. Two-tailed tests were used.

## Results

### Mass cytometry identifies the major leucocyte populations in murine atherosclerotic aortas

We used multi-parameter mass cytometry and high-dimensional analysis to examine the immune cell content of murine atherosclerotic aortas (see Supplementary material online, *Figure S1B*). Single-cell suspensions of aortas from ApoE^−^^/^^−^ mice fed either a chow or high fat diet were stained with a panel of 35 antibodies (see Supplementary material online, *Table S1*). Almost 40% of the live single cells acquired were CD45 positive (CD45^+^) (see Supplementary material online, *Table S2*). The CD45 negative (CD45^−^) cells included CD31 and alpha smooth muscle actin-positive cells (see Supplementary material online, *Figure S1B*), which are likely to be endothelial and smooth-muscle cells from the vessel wall.

**Figure 1:**
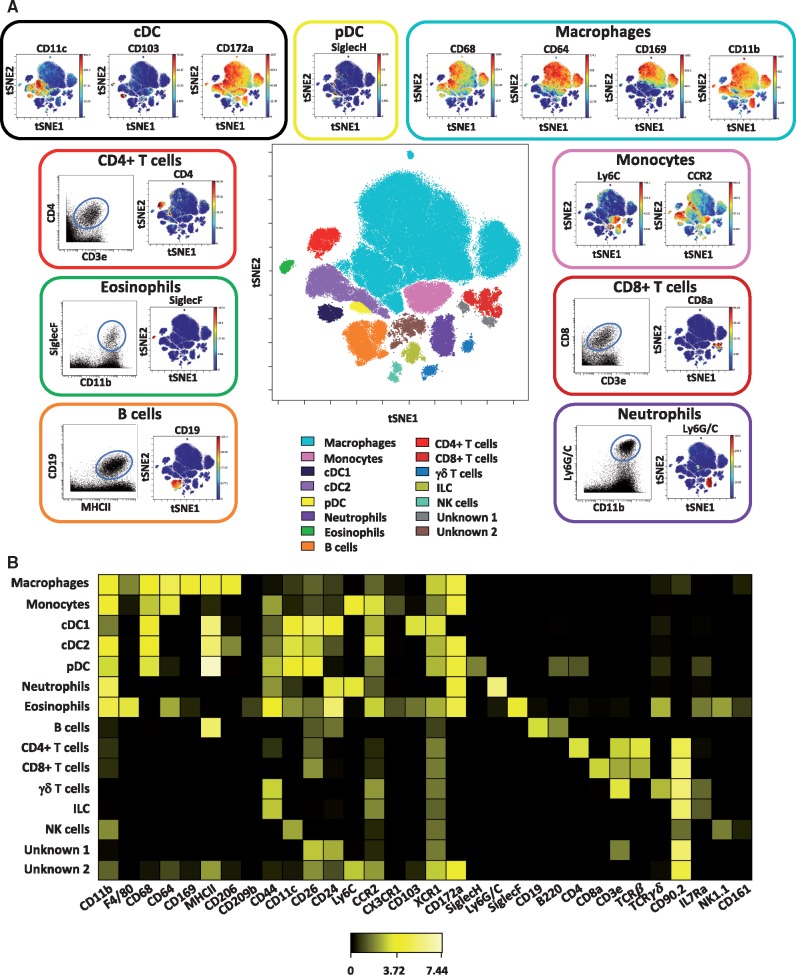
High-dimensional characterization of leucocyte populations in murine atherosclerotic aortas by mass cytometry. Single*-*cell suspensions of aortas from ApoE^−/−^ mice fed either a chow or high fat diet were stained with a panel of 35 antibodies. For each sample, cells from two aortas were pooled. *(A)* Live CD45^+^ cells concatenated from the aortas of all ApoE^−/−^ mice studied (both chow and high fat fed) (*n* = 13) were clustered using viSNE on expression of 35 cell surface and intracellular markers outlined in see Supplementary material online, *Table S1*. The analysis identifies 15 populations including myeloid, lymphocyte, and unknown subsets (centre plot). The selected populations are displayed in a viSNE dot plot showing the expression level of their major markers with or without a representative dot plot showing two relevant cell population markers (outer plots). *(B)* Heatmap showing the relative expression level of 32 cell markers within the 15 cell subsets identified by the viSNE clustering shown in (*A*).

To gain a broad overview of the immune cell populations present in the atherosclerotic aorta, we first performed a viSNE analysis on live CD45^+^ events concatenated from all ApoE^−^^/^^−^ mice studied, i.e. both chow and high fat diet fed*.* On the basis of marker expression, we identified at least 13 leucocyte populations, including major myeloid and lymphoid cell subsets, which accounted for over 95% of the total live CD45^+^ cells in the atherosclerotic mouse aorta (*Figure 1A* and see Supplementary material online, *Figure S2*). The most represented leucocyte subset was macrophages (defined as CD68^+^CD11b^+^CD64^+^Ly6C^−^), accounting for over 50% of the total CD45^+^ events. Other myeloid cell populations were monocytes (CD11b^+^Ly6C^+^CCR2^+^), neutrophils (CD11b^+^Ly6G/C^+^), and eosinophils (CD11b^+^SiglecF^+^) (*Figure 1A*). In addition to a small population of plasmacytoid dendritic cells (pDC) (SiglecH^+^B220^+^), two subsets of conventional dendritic cells (cDC) were found. cDC1 cells were CD11b^low^CD11c^+^MHCII^hi^CD172a^-^CD103^+^ whereas cDC2 cells (CD11b^+^CD11c^+^MHCII^hi^CD172a^+^) expressed CD172a and higher levels of CD11b.

Multiple lymphoid cell subsets were also present including CD19^+^ B cells, CD4^+^ T cells (TCRß^+^CD3^+^CD4^+^), CD8^+^ T cells (TCRß^+^CD3^+^CD8^+^), γδ^+^ T cells (TCRγδ^+^CD3^+^), NK cells (NK1.1^+^), and innate lymphoid cells (ILC; Lin^-^CD90.2^+^IL7Ra^+^). We could identify 13 subsets within the live CD45^+^ CD90.2^+^ CD3^+^ gated cells (see Supplementary material online, *Figure S3A–C*). These included three subsets of CD4^+^ T cells, four subsets of CD8^+^ T cells, and two subsets of γδ T cells. These subsets likely include memory, regulatory, and effector subsets. A paucity of B cell markers in our staining panel made discrimination of different subsets in viSNE analysis difficult. However, B cells expressed varying levels of multiple markers including B220, CD44, Ly6C, and CD24 (see Supplementary material online, *Figure S4*). In addition to these identifiable populations, the analysis also revealed subsets that were not fully interpretable on the basis of expression of the markers in our staining panel (*Figure 1B*).

### High fat feeding alters immune cell composition of the ApoE^−/−^ mouse aorta

**Figure 2:**
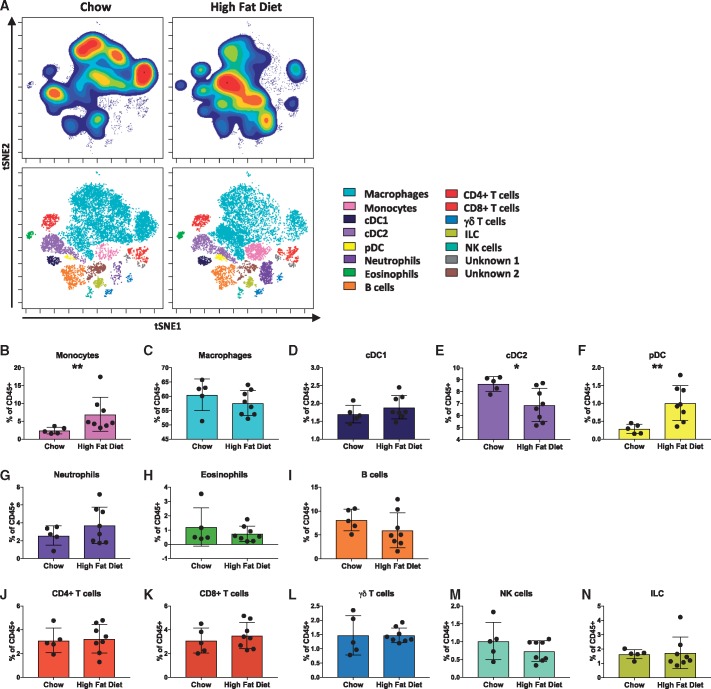
High fat feeding alters the immune cell composition of ApoE^−/−^ mice aortas. Single*-*cell suspensions of aortas from ApoE^−/−^ mice fed either a chow or high fat diet were stained with a panel of 35 antibodies. For each sample, cells from two aortas were pooled. *(A)* viSNE plots of clustered CD45^+^ leucocytes are displayed for representative chow and high fat diet fed ApoE^−/−^ mice, showing cell density of the population clusters. *(B*–*N)* Bar graphs showing the changes in abundance of the cell populations identified in the viSNE clustering outlined in *Figure 1*, between chow and high fat diet fed mice: monocytes (*B*), macrophages (*C*), conventional type 1 dendritic cells (cDC1) (*D*), conventional type 2 dendritic cells (cDC2) (*E*), pDC (*F*), neutrophils (*G*), eosinophils (*H*), B cells (*I*), CD4^+^ T cells (*J*), CD8^+^ T cells (*K*), γδ T cells (*L*), natural killer (NK) cells (*M*), and ILC) *(N*) Data are presented as mean± SD. Dots represent individual samples, *n* = 5–8, *P < 0.05, **P < 0.01.

Feeding ApoE^−^^/^^−^ mice a high fat diet accelerates atherosclerotic lesion development resulting in the formation of more advanced lesions^17^. We examined aortas from ApoE^−^^/^^−^ mice fed a chow or high fat diet, to determine whether diet induces changes in leucocyte subset abundance. No difference in the percentage of total live cells or live CD45^+^ cells was observed between the two groups (see Supplementary material online, *Table S2*) and all of the populations described in the concatenated sample were present in mice fed either diet (*Figure 2A*). However, compared to chow-fed ApoE^−^^/^^−^ mice, aortas from ApoE^−^^/^^−^ mice fed a high fat diet had increased monocytes (*Figure 2B*) and pDC (*Figure 2F*). In contrast, cDC2 were decreased (*Figure 2E*) in the aortas of high fat compared to chow fed ApoE^−^^/^^−^ mice. Although statistical significance was not reached, a trend toward increased neutrophils (*Figure 2G*) and reduced B cells (*Figure 2I*) was observed in aortas of high fat compared to chow fed ApoE^−^^/^^−^ mice. There was a significant reduction in TCRβ^+^Ly6C^+^CCR2^+^CD26^+^CD44^+^ T cells between chow and high fat fed ApoE^−^^/^^−^ mice (see Supplementary material online, *Figure S3D*). No differences in the proportion of other T cell or lymphoid subsets was observed.

### Altered aortic macrophage populations in ApoE^−/−^ mice fed a high fat diet

**Figure 3:**
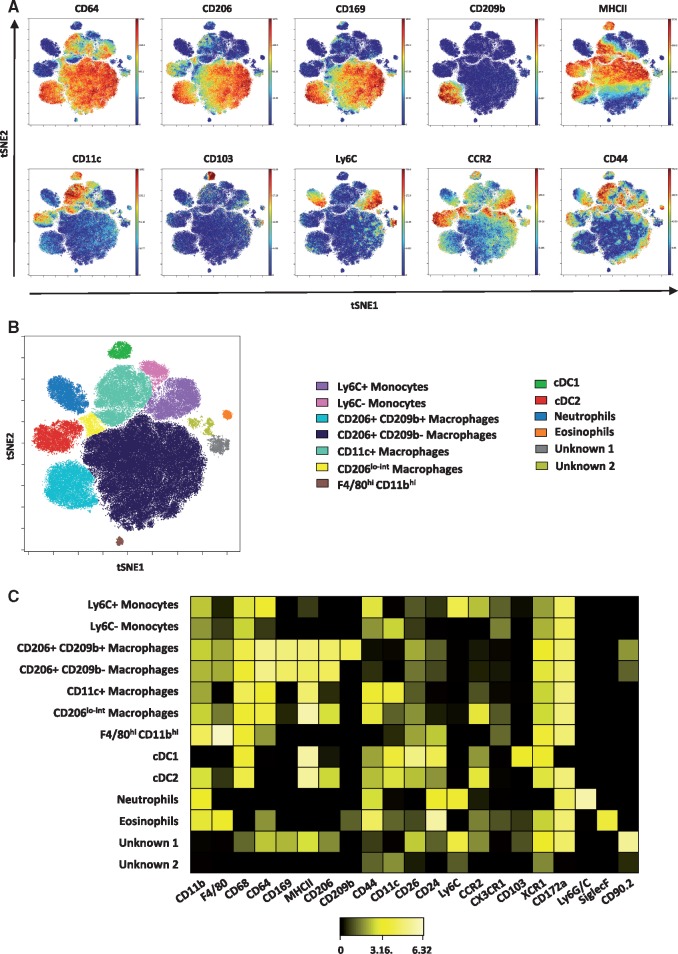
Mass cytometry reveals five macrophage subsets in the atherosclerotic aorta. *(A*) Myeloid cells were gated as Lin-CD11b^lo-hi^ concatenated from the aortas of all ApoE^−/−^ mice studied (*n* = 13) and clustered using viSNE on the expression of 21 cell surface and intracellular markers outlined in see Supplementary material online, *Table S1*. Expression levels of selected myeloid markers in the resulting viSNE clustered cell populations is shown. *(B)* 13 cell populations consist of monocytes (Ly6C^+^ and Ly6C^−^), conventional type 1 and type 2 dendritic cells (cDC1 and cDC2), granulocytes (neutrophils and eosinophils), five macrophage subsets and two unidentified populations. *(C)* Heatmap showing the relative expression level of 21 cell markers within the 13 myeloid cell subsets identified by the viSNE clustering shown in (*B*).

Myeloid cells (neutrophils, eosinophils, monocytes, macrophages, and dendritic cells) are known to play important roles in atherosclerosis development. Myeloid cells (gated as Lin^-^CD11b^lo-hi^) accounted for around 75% of the CD45^+^ leucocytes in the ApoE^−^^/^^−^ aortas in our study. Application of the viSNE algorithm to the concatenated myeloid cells of all ApoE^−^^/^^−^ mice studied and gating based on distribution of marker expression (*Figure 3A*) enabled the identification of subsets of macrophages and monocytes that were not as easily determined in the analysis of all the CD45+ cells. Indeed, at least 13 myeloid cell subsets were found (*Figure 3B*). The heatmap (*Figure 3C*) displays how marker expression and intensity differs between the subsets. In addition to neutrophils, eosinophils and cDC1 and cDC2 subsets that were detected in the total CD45^+^ cell analysis, focused analysis of the myeloid populations revealed two monocyte, five macrophage, and two unknown subsets. The unknown subsets accounted for less than 3% of the total myeloid cells.

The monocyte subsets included a CCR2^hi^Ly6C^+^ population and a Ly6C^-^CCR2^-^CX3CR1^+^ population. The majority of the aortic macrophages were CD206^+^ CD169^+^ and could be further separated into two subsets on the basis of CD209b expression. The third most abundant population of macrophages was a CD11c^+^ subset, while a fourth macrophage subset was identifiable as CD206^lo-int^. Additionally, a small population of F4/80^hi^ CD11b^hi^ macrophages was also observed.

**Figure 4:**
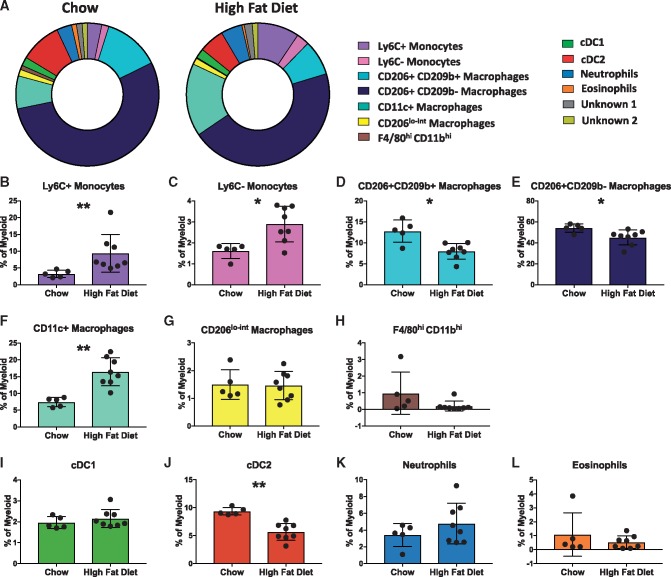
High fat feeding reshapes the myeloid cell composition of murine atherosclerotic aortas. *(A)* Doughnut plots show the proportions of the 13 myeloid cell populations from the viSNE analysis in the aortas of chow and high fat diet fed ApoE^−/−^ mice. *(B–L)* Bar graphs showing the changes in abundance of the cell populations identified in the viSNE clustering outlined in *Figure 3*, between chow and high fat diet fed mice: Ly6C^+^ monocytes (*B*), Ly6C^−^ monocytes (*C*), CD206^+^ CD169^+^CD209b^+^ macrophages (*D*), CD206^+^CD169^+^CD209b^−^ macrophages (*E*), CD11c^+^ macrophages (*F*), CD206^lo-int^ macrophages (*G*), F4/80^hi^ CD11b^hi^ macrophages (*H*), conventional type 1 dendritic cells (cDC1) (*I*), conventional type 2 dendritic cells (cDC2) (*J*), neutrophils (*K*), and eosinophils (*L*). Data are presented as mean± SD. Dots represent individual samples, *n* = 5–8 *P < 0.05, **P < 0.01.

Next, we assessed whether high fat feeding of ApoE^−^^/^^−^ mice alters the aortic myeloid cell composition. No difference in the percentage of myeloid cells (as a percentage of CD45^+^ cells) was observed between the aortas of chow and high fat fed ApoE^−^^/^^−^ mice (see Supplementary material online, *Table S3*). However, the proportion of several myeloid cell subsets between the two groups was significantly different (*Figure 4A*). In the aortas of high fat fed ApoE^−^^/^^−^ mice, there was a striking increase in the proportion of both monocytes (Ly6C^+^ and Ly6C^-^ subsets) and CD11c^+^ macrophages compared to aortas from chow fed animals (*Figure 4B, C,* and *F*). Conversely, the proportions of CD206^+^ CD169^+^CD209b^−^ macrophages, CD206^+^CD169^+^CD209b^+^ macrophages, and cDC2 cells were reduced in the aortas of ApoE^−^^/^^−^ fed a high fat diet (*Figure 4D, E,* and *J*).

**Figure 5:**
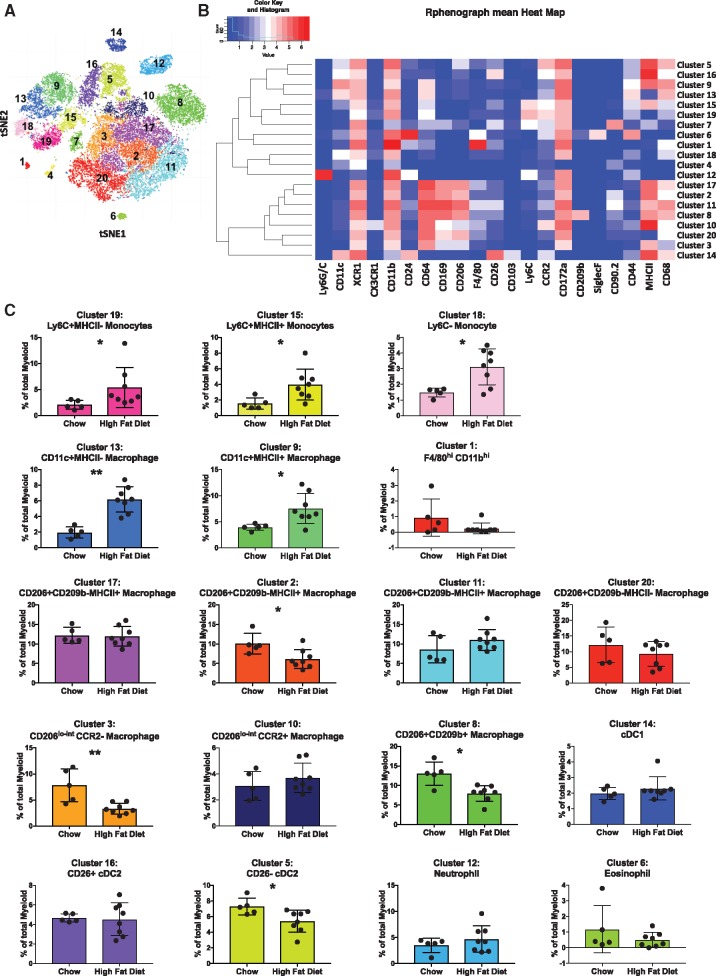
Unbiased multi-dimensional analysis by CyTOFkit Phenograph reveals 20 myeloid cell populations in murine atherosclerotic aortas. *(A)* Files containing the myeloid-gated cells used for the viSNE clustering in *Figure 3* were exported from Cytobank into R. Myeloid cells were clustered on the same cell markers as the viSNE analysis in *Figure 3* using Phenograph, an element of the Cytofkit Bioconductor package. Shown is the resulting t-SNE plot highlighting the 20 cell clusters identified by phenograph. (*B)* Heatmap showing the expression levels of 21 myeloid markers in the 20 identified cell clusters (*C)*. 18 of the 20 cell clusters are identified by their marker expression levels and the changes in abundance of each population between chow and high fat diet fed mice is shown. The analysis sub-divides CD206^+^ CD169^+^CD209b^−^ MHCII^+^ macrophages into three clusters by the expression of CD90.2, CD26, F4/80, and CD68. CD11c^+^ macrophages and Ly6C+ monocytes are separately into two clusters by MHCII expression. Conventional type type 2 dendritic cells (cDC2) are also divided into two clusters by CD26. Data are presented as mean ± SD. Dots represent individual samples, *n* = 5–8 *P < 0.05, **P < 0.01.

To confirm the myeloid subset identification and avoid reliance on gating for identification, we used Phenograph in CyTOFKit to enable automatic detection of cell populations in an unbiased manner ^14^. Phenograph analysis revealed 20 myeloid cell clusters (*Figure 5A*). A heatmap of the expression of each marker by each cluster is shown in *Figure 5B*. By comparing the expression of the markers between clusters, we were able to identify them and the majority aligned with the populations identified through viSNE (*Figure 3B*).

**Figure 6:**
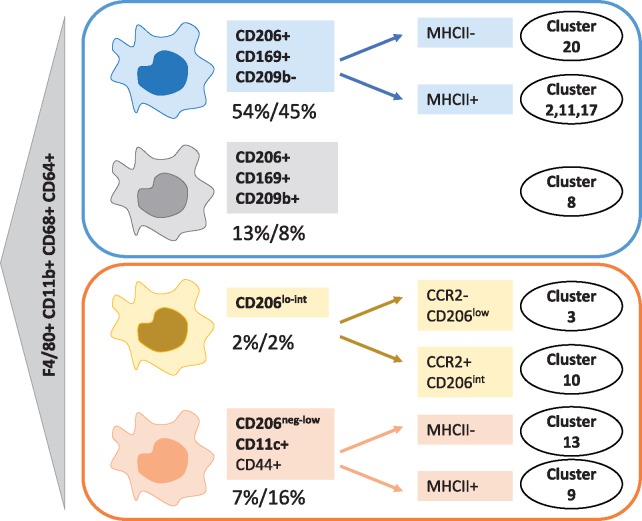
Schematic diagram showing macrophage subsets identified in aortas of ApoE^−/−^ mice. viSNE and phenograph analysis of Lin-CD11b^lo-hi^ cells from ApoE^−/−^ mice fed a chow and high fat diet revealed the presence of four subsets of macrophages. Percentages shown represent the average proportion of each subset in aortas of either chow (left number, *n* = 5) or high fat (right number, *n* = 8) fed ApoE^−/−^ mice. Where phenograph could identify multiple clusters within a subset the main differences between the clusters is shown. Macrophage cluster numbers assigned by phenograph (*Figure 5*) are shown against each subset/cluster.

Phenograph determined two clusters of Ly6C^+^ monocytes based on MHCII expression (*Figure 5*). Three out of five macrophage subsets were found to comprise further cellular communities. CD206^+^ CD169^+^CD209b^−^ macrophages were the most heterogenous subset and they housed four clusters (clusters 2, 11, 17, and 20) (*Figure 5B*). Cluster 20 was negative for MHCII whereas clusters 2, 11, and 17 were MHCII^+^. Clusters 2, 11, and 17 were almost identical bar subtle differences in CD68 expression (*Figure 5B*). Cluster 2 was significantly under-represented in aortas of high fat compared to chow fed ApoE^−^^/^^−^ mice (*Figure 5C*). Two clusters of CD206^lo-int^ macrophages were differentiated by phenograph (cluster 3 and cluster 10). Cluster 10 was CCR2^+^CD169^int^ whereas cluster 3 was CCR2^-^CD169^−^ and was significantly reduced in aortas of high fat fed ApoE^−^^/^^−^ mice. In phenograph, the proportion of CD206^+^ CD169^+^ macrophages (cluster 8) was also significantly reduced in aortas of high fat ApoE^−^^/^^−^ mice, confirming the viSNE result (*Figure 5C*). Two CD11c^+^ macrophage clusters (differing in MHCII expression) were identified and both were significantly increased with high fat feeding (*Figure 5C*). Phenograph additionally identified neutrophils, eosinophils, cDC1, and two clusters of cDC2 cells (CD26^+^ and CD26^−^). CD26^−^ cDC2 cells (cluster 5) were significantly reduced in high fat fed ApoE^−^^/^^−^ aortas. Macrophage subsets and clusters revealed by tSNE and phenograph are summarized in *Figure 6*.

## Discussion

Differences in ontogeny, location, and activation status drive the generation of diverse mononuclear phagocytes in mammalian tissues^18^. Vascular myeloid cells are poorly resolved when compared to their lung, cardiac, brain, liver, and gut equivalents. Here, we describe how mass cytometry can be used to perform high-parameter analysis of leucocyte subsets in the atherosclerotic murine aorta.

For many years, the mainstay of immune cell phenotyping in atherosclerotic plaques has been immunohistochemistry. Despite the considerable impact this technique has had on the field, immunohistochemical staining for more than three to four different antigens is technically challenging. The application of flow cytometry to the aorta^11^^,^^12^, allowing simultaneous detection of around eight antigens, was a major technological step forward. Although 12–18 parameter flow cytometry is now commonplace, significant challenges, in particular with tissue auto-fluorescence and spectral overlap, remain. Mass cytometry offers the next significant advance in the field of atherosclerosis research as it allows high-dimensional multi-parameter (currently up to 40 parameter) analysis at the single cell level.

Dendritic cells (DC) are important bridges between innate and adaptive immunity through antigen presentation to T cells and Treg homeostasis. The nomenclature of DC subsets is complex, however a unified system was proposed, which separates DC into three main subsets: type 1 conventional dendritic cells (cDC1), cDC2, and pDC^19^. DC are present in the vascular wall and in atherosclerosis are versatile and have many roles including lipid uptake, efferocytosis, and cytokine production^20^^,^^21^. cDC1 are considered athero-protective via Treg induction whereas a subset of CCL17-expressing DC has been identified as pathogenic^22^^,^^23^. The role of pDC in atherosclerosis is a matter of debate^12^^,^^24^.

Guilliams *et al.*, recently described a set of markers for identification of dendritic cells as part of two subsets: cDC1 and cDC2, across tissues and species^25^. This set of markers has never been applied to vascular tissues. Using this approach we were able to clearly identify cDC1 and cDC2 in the murine atherosclerotic aorta and resolve them from the macrophage populations. This permitted the elucidation of reciprocal changes induced by the pro-atherogenic diet between macrophages and dendritic cells. We showed that when ApoE^−^^/^^−^ mice are fed a high fat diet, cDC1 remain unchanged whereas cDC2 arterial content is decreased and pDCs are significantly increased. Thus, our study provides a toolbox of markers that can be reliably used to discriminate dendritic cells from other myeloid populations and to monitor their atherosclerosis associated changes in the vasculature.

Macrophage identification in tissue is challenging as they display a continuum of phenotypes in response to environmental conditions. Several commonly used markers are not as selective as once thought. Expression of the scavenger receptor CD68 is acquired by non-myeloid cells in the intima during atherosclerosis development^26^. Our analysis shows that CD68 expression is found on all monocyte, macrophage and dendritic cell subsets in the murine aorta, while it was not expressed by lymphocytes, neutrophils, and eosinophils. Hence, CD68 expression in the aorta is not a reliable marker for macrophage identification if used in isolation. The mannose receptor, CD206, is a marker of alternatively-activated or ‘M2-like’ macrophages^27^. However, our data indicate that in the context of murine atherosclerosis, it is highly expressed by the majority of aortic macrophages. Thus, there is an urgent need for reliable markers to identify and qualify macrophages within lesions. CD64 was proposed by the ImmGen Consortium as a potential differentiator between murine macrophages and dendritic cells in the steady state^28^. Its usefulness in pathological settings is still being debated. In agreement with other tissues^28^^,^^29^, we demonstrate that CD64 (Fc gamma receptor 1) can help identify aortic macrophages and discriminate them from dendritic cells and eosinophils, the latter expressing high levels of F4/80.

Mass cytometry followed by clustering algorithms revealed that at least five subsets of macrophages exist in the murine atherosclerotic aorta (*Figure 6*). The majority of the aortic macrophages bear markers usually associated with resident macrophages and were identifiable as CD206^+^ CD169^+^ and could be further separated into two distinct subsets on the basis of CD209b expression. The third most abundant population of macrophages was a CD11c^+^ subset of macrophages. A fourth macrophage subset was identifiable as CD206^lo-int^. The fifth was the smallest subset with a distinct F4/80^hi^ CD11b^hi^ phenotype. Due to their levels being close or below 1% of the myeloid cells this fifth subset might represent a contamination with peri-adventitial fat from the aorta^30^ or serosal macrophages^18^. Phenograph delineated further clusters within these macrophage subsets mainly on the basis of MHCII and CCR2 expression (summarized in *Figure 6*). In the heart and the aorta, embryonically derived MHCII^−^ macrophages are progressively replaced by MHCII^+^ counterparts in adulthood^7^^,^^31^. However, the functional implications of the segregation between MHCII^+^ and MHCII^−^ clusters remains to be determined.

We show that high fat feeding leads to a shift in the proportion of selected macrophage subsets (summarized in see Supplementary material online, *Figure S5*). We recently described that IRF5 drives expression of CD11c, a hallmark of a subset of macrophages with a pro-inflammatory role in atherosclerosis^32^. IRF5 deletion reduces the formation of the necrotic core by enhancing the expression of efferocytic receptors and efferocytosis^32^. Tacke *et al.* reported that CD11c^+^ myeloid cells could be found in proximity to the necrotic core in atherosclerotic lesions^33^. In this study, we show that this CD11c^+^ macrophage subset is expanded with high fat feeding in ApoE^−^^/^^−^ mice providing further support to the idea that these macrophages are integral to detrimental plaque inflammation. This macrophage subset also expressed high levels of CD44. CD44, a receptor known to be involved in cell adhesion and migration, has been recently reported to help identify recruited myeloid subsets from the resident microglia in the murine brain^34^. Our findings support a similar phenomenon in the vascular compartment. The aortas of ApoE^−^^/^^−^ mice fed a high fat diet also contained a higher proportion of monocytes compared to the aortas of chow fed animals, as previously shown^35^.

Concomitantly, we observed a reduction in the proportion of both the CD209b^+^ and CD209b^−^ subsets of CD206^+^ CD169^+^ macrophages with high fat feeding, suggesting that high fat diet consumption selects inflammatory mono-macrophage subsets over resident macrophage subsets. Detection of these perturbation-induced changes are only possible with a multi-parametric platform that simultaneously monitors several immune cell populations at the single cell level. The use of mass cytometry and the panel of antibodies validated in this study elucidate in unprecedented detail how macrophage populations are shaped by lesion progression and offer invaluable tools to further interrogate the biology of these macrophage subsets and how they change following perturbations.

A caveat of our study is that we cannot exclude contamination of our samples with non-aortic cells, deriving for instance from the thymus. Due to the anatomy of the aorta and the relatively small numbers of immune cells contained within it, dissection techniques need refinement to avoid carry over from other organs during dissection. Despite the significant technological advance that CyTOF brings to studying murine atherosclerotic tissue, it does not provide positional information, which may contribute to a particular cell phenotype. Imaging mass cytometry^36^ combining CyTOF and laser capture could be useful to address this issue. Our study validates a panel of antibodies that could be useful for such studies in the near future.

During revision of this paper, Winkels *et al.* published a study containing single-cell RNA sequencing (scRNASeq) and CyTOF data in murine atherosclerotic aortas^37^. In the same issue, Cochain *et al.* published scRNASeq on murine aortas^38^. Different markers were used for the clustering algorithms in our manuscript and the one of Winkels *et al.*, which makes direct comparison between the subsets reported in the two studies challenging. The panel used by Winkels *et al.*^37^ is comparatively more skewed toward lymphocyte markers and could identify B cell subsets in greater detail than our panel. Phenograph analysis by Winkels *et al.* revealed 10 myeloid cell clusters whilst our study, which had a bias toward myeloid cell markers, identified 20 clusters and provided superior resolution of monocytes, dendritic cell, and macrophage subsets.

An additional contributing factor to the variations is the modality of cell isolation from murine aortas. For instance, Winkels *et al.* report the percentage of macrophages to be 7–18% of CD45^+^ cells in the CyTOF data set and 5–10% of CD45^+^ cells in the scRNASeq data^37^, while in our study, macrophages comprise at least 50% of the aortic CD45^+^ cells, which is closer to the proportion observed by Cochain *et al.* in LDLR^−^^/^^−^ mice^38^. These disparities may result from different cell isolation techniques, with a preferential loss of monocyte-macrophages as acknowledged by the authors after genetic deconvolution analysis in bulk aorta transcriptomes. Thus, our data and that of Winkels *et al.* and Cochain *et al.*^37^^,^^38^ provide complementary knowledge on leucocytes in atherosclerosis. We believe that our study and the concomitant papers herald the interesting times ahead where we can apply new technology to have a ‘look in’ the plaque as never before.

CyTOF mass cytometry is a step change in the field of CVD. Along with the rapid recent technological improvements in scRNASeq and multi-parameter flow cytometry, the three technologies significantly improve our ability to phenotype heterogeneous cell samples such as those from atherosclerotic tissues. The advantages that CyTOF brought to this particular study included the ability to undertake multi-parameter phenotyping of cell surface and intracellular protein expression on the entire cell population isolated from murine aortas in the absence of any constraints on staining panel size and design due to autofluorescence. The ease of custom antibody conjugation also allowed us enormous flexibility in staining panel design. Advantages and limitations of mass cytometry compared to other single-cell techniques have been reviewed in (10).

We envisage that CyTOF mass cytometry will become the method of choice for analysis of immune cell subsets in both murine and human vascular tissues, where tissue autofluorescence and limited cell numbers are key challenges. It could be applied to phenotyping of arterial health and pathology. Sample size required for robust data will vary depending on the experimental conditions being examined^34^^,^^37^^,^^39^^,^^40^. In our myeloid cell analysis, 21 markers were used for clustering (see Supplementary material online, *Table S1*). Our panel and the nomenclature that it proposes for myeloid cells in the murine aortic lesions is a useful guide to revise what we know about immune cell populations in the murine aorta.

Development of novel therapeutics targeting specific aspects of the inflammatory component of atherosclerosis remains a key goal. Due to their complexity, macrophages have not yet been therapeutically targeted in atherosclerosis. Increased understanding of the cellular composition of diseased tissue and the function of specific cellular subsets will enable the development of better, more selectively targeted therapies. Mass cytometry may provide the means to realize this goal. Impervious to autofluorescence and able to resolve small cell subsets, CyTOF could represent the new gold standard to decipher the immune cell landscape in CVD.

## Supplementary material

Supplementary material is available at *Cardiovascular Research* online.

**Conflict of interest:** none declared.

## Funding

This work was supported by funding from the European Community's Seventh Framework Programme [FP7-2007-2013] under grant agreement n°HEALTH-F2-2013-602114 (Athero-B-Cell) and The Kennedy Trust for Rheumatology Research. PM is supported by the British Heart Foundation grants PG/12/81/29897 and RE/13/5/30177.

## Supplementary Material

Supplementary Data
