# Supplementary material for: Immune cell census in murine atherosclerosis: cytometry by time of flight illuminates vascular myeloid cell diversity
Source: Cardiovasc Res. 2018 May 2;114(10):1360–71. doi: 10.1093/cvr/cvy109 (PMC6054192; doi:10.1093/cvr/cvy109)
Supplement: Supplementary Data [file cvy109_cole_et_al_cvr-2018-299_supplementary.pdf]

CVR-2018-299

Supplemental Material

Immune cell census in murine atherosclerosis: cytometry by time of flight illuminates vascular myeloid cell diversity

Jennifer E Cole[1]*, Inhye Park[1]*, David Ahern[1], Christina Kassiteridi[1], Dina Danso Abeam[1], Michael Goddard[1], Patricia Green[1], Pasquale Maffia[2,3,4], Claudia Monaco[1]

[1]Kennedy Institute of Rheumatology, Nuffield Department of Orthopaedics, Rheumatology and Musculoskeletal Sciences, University of Oxford, Oxford, United Kingdom;

[2]Centre for Immunobiology, Institute of Infection, Immunity and Inflammation, College of Medical, Veterinary and Life Sciences, University of Glasgow, Glasgow, United Kingdom;

[3]Institute of Cardiovascular and Medical Sciences, College of Medical, Veterinary and Life Sciences, University of Glasgow, Glasgow, United Kingdom;

[4]Department of Pharmacy, University of Naples Federico II, Naples, Italy.

* These authors contributed equally

Corresponding author

Professor Claudia Monaco

Kennedy Institute of Rheumatology

Roosevelt Drive

Headington

Oxford, OX3 7FY

United Kingdom

E-mail:claudia.monaco@kennedy.ox.ac.uk

Tel:+44 1865 612636

Fax:+44 1865 612601

Supplemental Table 1: List of antibody clones used in CyTOF analysis

Metal Label	Antigen	Clone	Manufacturer	Conjugation	Figures used in for clustering
**141Pr**	Ly-6G/C (Gr-1)	RB6-8C5	Fluidigm	Fluidigm	1-3, 5
**142Nd**	CD11c	N418	Fluidigm	Fluidigm	1-3, 5
**143Nd**	IL7Ra	A7R34	Biolegend	In-house	1,2, S2, S3
**144Nd**	XCR1	ZET	Biolegend	In-house	1-3, 5
**145Nd**	TCRgd	GL3	Biolegend	In-house	1, 2, S2
**146Nd**	CX3CR1	SA011F11	Biolegend	In-house	1-3, 5, S3
**147Sm**	CD45	30-F11	Fluidigm	Fluidigm	None
**148Nd**	CD11b (Mac-1)	M1/70	Fluidigm	Fluidigm	1-3, 5
**149Sm**	CD19	6D5	Fluidigm	Fluidigm	1, 2
**150Nd**	CD24	M1/69	Fluidigm	Fluidigm	1-3, 5, S3
**151Eu**	CD64	X54-5/7.1	Fluidigm	Fluidigm	1-3, 5
**152Sm**	CD3e	145-2C11	Fluidigm	Fluidigm	1, 2
**153Eu**	CD335(NKp46)	29A1.4	Fluidigm	Fluidigm	1, 2
**154Sm**	CD31 (PECAM-1)	390	Abcam	In-house	None
**155Gd**	ACTA 2	1A4	Sigma	In-house	None
**156Gd**	CD169	3D6.112	Biolegend	In-house	1-3, 5
**158Gd**	CD206	C068C2	Biolegend	In-house	1-3, 5
**159Tb**	F4/80	BM8	Fluidigm	Fluidigm	1-3, 5
**160Gd**	CD26	DPP-4	Biolegend	In-house	1-3, 5, S2, S3
**161Dy**	CD103	2E7	Biolegend	In-house	1-3, 5, S2, S3
**162Dy**	Ly6C	HK1.4	Fluidigm	Fluidigm	1-3, 5, S2, S3
**163Dy**	CCR2	SA203G11	Biolegend	In-house	1-3, 5, S2, S3
**164Dy**	CD172a	SIRPa	Biolegend	In-house	1-3, 5, S2, S3
**165Ho**	CD161 (NK1.1)	PK136	Fluidigm	Fluidigm	1, 2, S2
**166Er**	CD209b (SIGNR1)	2C7B27	Biolegend	In-house	1-3, 5
**167Er**	SIGLECF	E50-2440	BD	In-house	1-3, 5
**168Er**	CD8a	53-6.7	Fluidigm	Fluidigm	1, 2, S2
**169Tm**	TCRb	H57-597	Fluidigm	Fluidigm	1, 2, S2
**170Er**	CD90.2	30-H12	Biolegend	In-house	1-3, 5
**171Yb**	CD44	IM7	Fluidigm	Fluidigm	1-3, 5, S2, S3
**172Yb**	CD4	RM4-5	Fluidigm	Fluidigm	1, 2, S2
**173Yb**	SIGLECH	551	Biolegend	In-house	1, 2
**174Yb**	I-A/I-E (MHCII)	M5/114.15.2	Fluidigm	Fluidigm	3, 5
**175Lu**	CD68	FA-11	Biolegend	In-house	1-3, 5
**176Yb**	CD45R (B220)	RA3-6B2	Fluidigm	Fluidigm	1, 2, S3

Supplemental Table 2: Baseline composition of aortas from chow and high fat diet fed ApoE-/- mice

Diet	Live (% of cells) mean $\pm$ SD	p (vs chow)	CD45+ (% of Live cells)	p (vs chow)
Chow	85.6 $\pm$ 3.9	-	35.1 $\pm$ 5.0	-
High Fat	87.2 $\pm$ 4.7	ns	38.4 $\pm$ 4.1	ns

Supplemental Table 3: Percentage of Myeloid and T cells

Diet	Myeloid (% of CD45+ cells) mean $\pm$ SD	p (vs chow)	CD90.2+CD3+ (% of CD45+ cells) mean $\pm$ SD	p (vs chow)
Chow	75.58 $\pm$ 5.0	-	7.97 $\pm$ 2.35	-
High Fat	76.35 $\pm$ 6.3	ns	8.49 $\pm$ 2.35	ns

Supplemental Figures and Legends

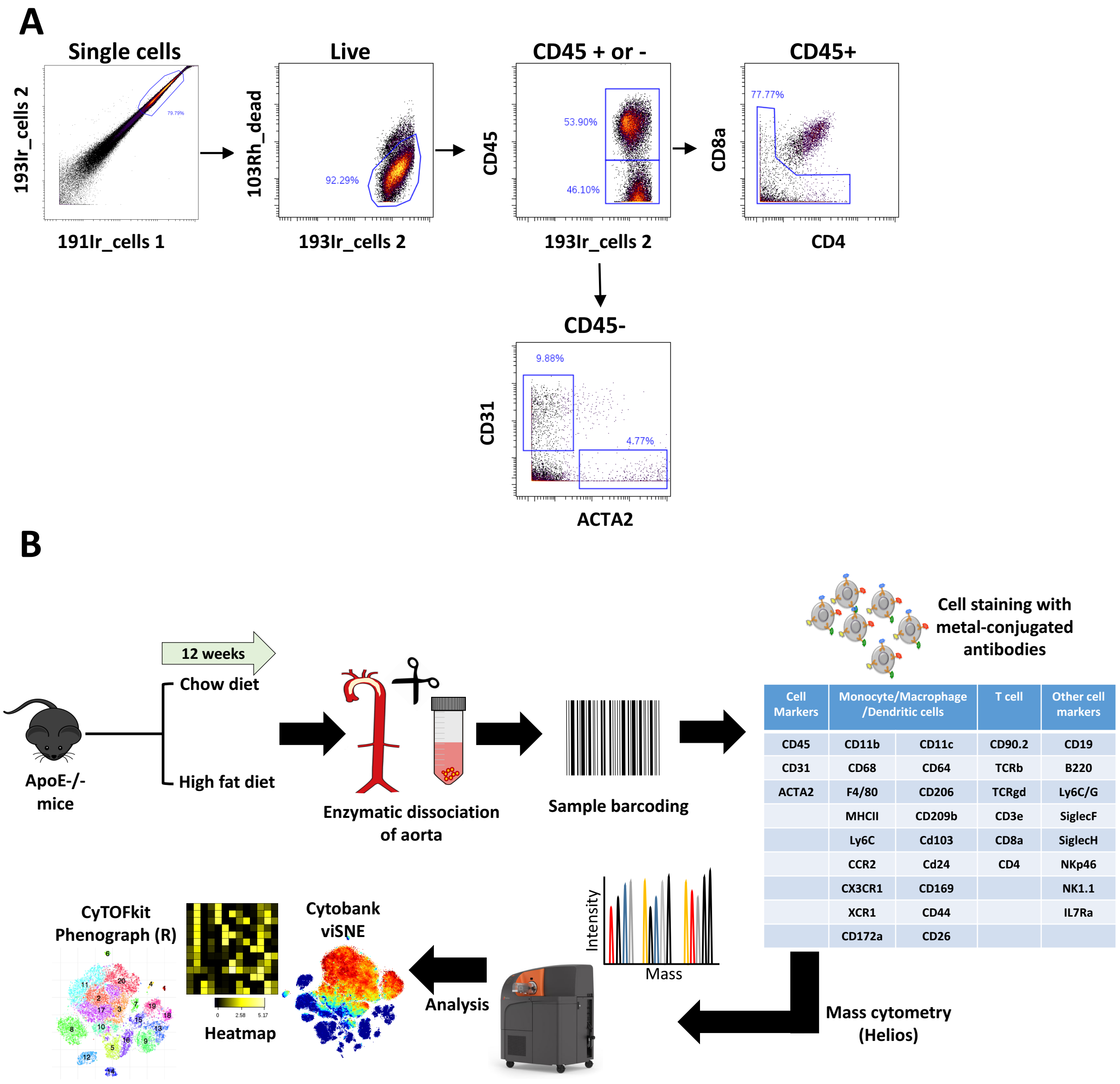

Figure S1 Gating strategy for Live CD45+ leucocytes and schematic illustration of experimental design. A. Iridium DNA intercalator double positive events were gated as cells and normalisation beads were removed. Rhodium DNA intercalator negative cells were gated as live cells, which were then separated by the expression of CD45. $CD45^{+}$ cells were regarded as leucocytes and $CD45^{-}$ populations were further gated by CD31 and alpha smooth muscle cell actin 2 (ACTA2). $CD45^{-}CD31^{+}$ and $CD45^{-}ACTA2^{+}$ cells can be identified as endothelial cells and smooth muscle cells, respectively. To reduce thymic contamination, $CD45^{+}CD4^{+}CD8^{+}$ populations were removed from analyses. **B** Apolipoprotein E-deficient (ApoE-/-) mice were fed a chow or high fat diet at the age of 12-week for 12 weeks. Aortas were collected from the mice and single cells obtained by enzymatic digestion. Cells then underwent barcoding and staining using a panel of 35 cell markers (supplemental table 1). Data from the stained cells were acquired using Helios and were analysed using Cytobank (viSNE and heatmap) and CyTOFkit phenograph.

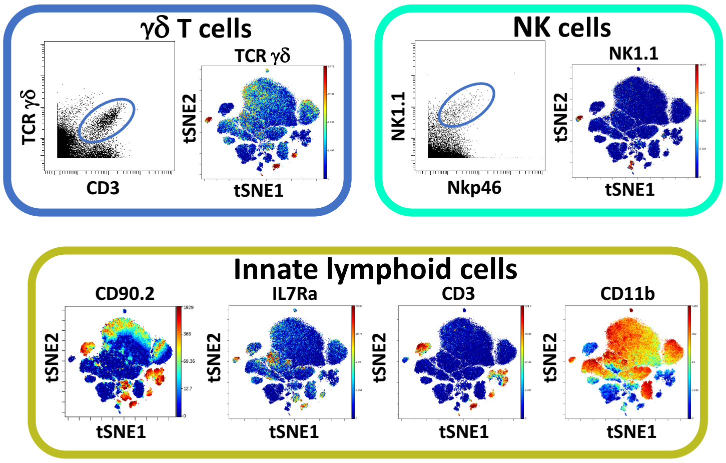

Figure S2 Identification of leucocyte populations in aortas of ApoE-/- mice. Single cell suspensions of aortas from Apolipoprotein E-deficient (ApoE-/-) mice fed either a chow or high fat diet were stained with a panel of 35 antibodies. For each sample, cells from 2 aortas were pooled. Live $CD45^+$ cells concatenated from the aortas of all apolipoprotein E-deficient (ApoE-/-) mice studied (both chow and high fat fed) (n=13) were clustered using viSNE on expression of 35 cell surface and intracellular markers outlined in supplemental table 1. The analysis identifies 15 populations including myeloid, lymphocyte and unknown subsets (Figure 1). The selected populations are displayed in a viSNE dot plot showing the expression level of their major markers with or without a representative dot plot showing two relevant cell population markers.

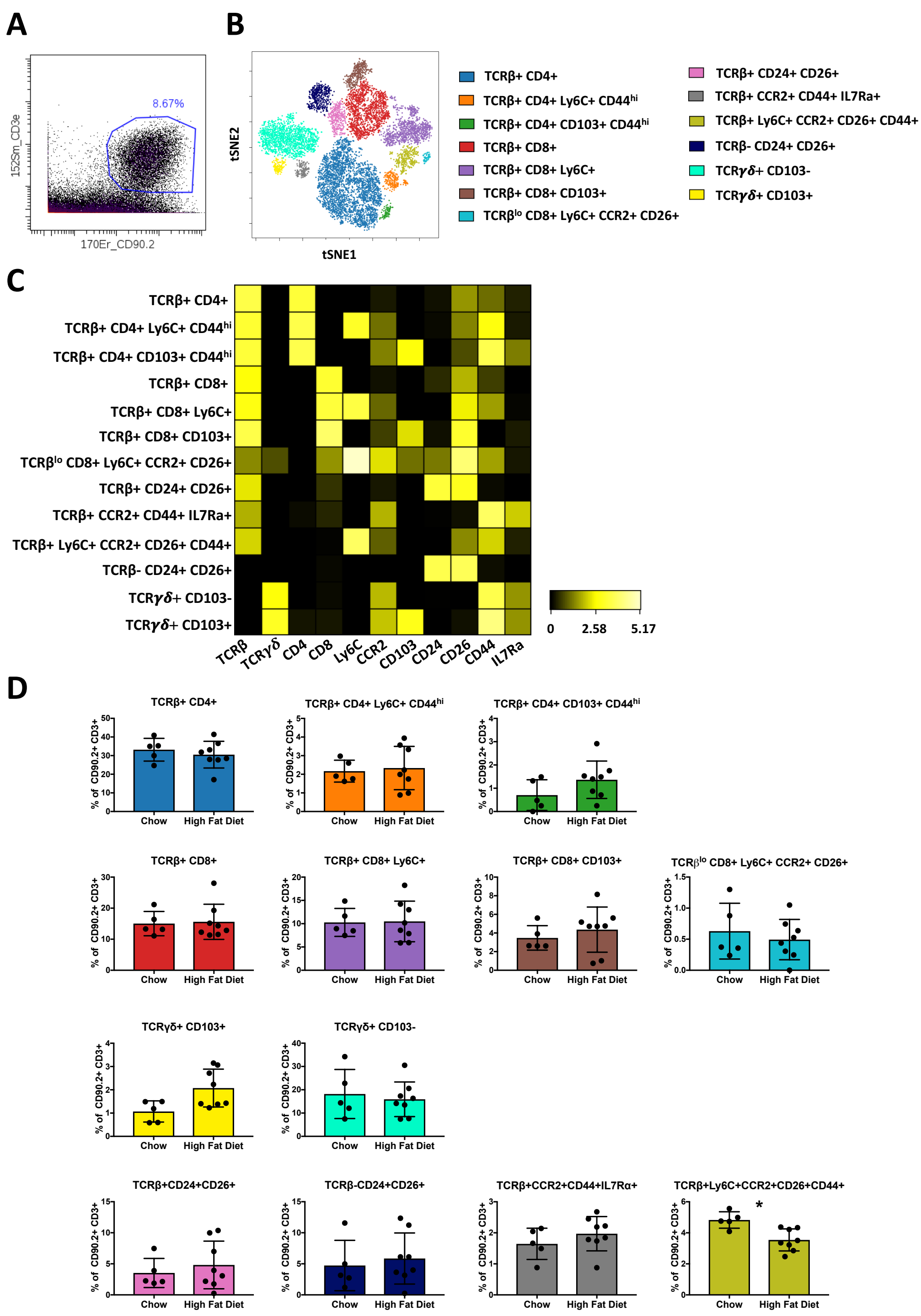

Figure S3 viSNE analysis reveals multiple T lymphocyte subsets in murine atherosclerotic aortas. A. Dot plot showing T cells gating (CD3e$^+$CD90.2$^+$ from live Lin$^-$CD45$^+$ leucocytes). **B.** T lymphocytes were clustered using viSNE on the expression of 12 cell surface and intracellular markers outlined in supplemental table 1. Identification of the 13 cell population clusters which included 3 CD4$^+$ T cells, 3 CD8$^+$ T cells, 2 $\gamma\delta$ T cells and 5 unknown populations. **C.** Heatmap showing the relative expression level of 11 cell markers within the 13 T cell subsets identified by the viSNE clustering shown in B. **D.** Bargraphs showing the changes in abundance of the cell populations identified in the viSNE clustering outlined in B, between chow and high fat diet fed mice. Data are presented as Mean± SD, Dots represent individual samples, n=5-8 *P<0.05

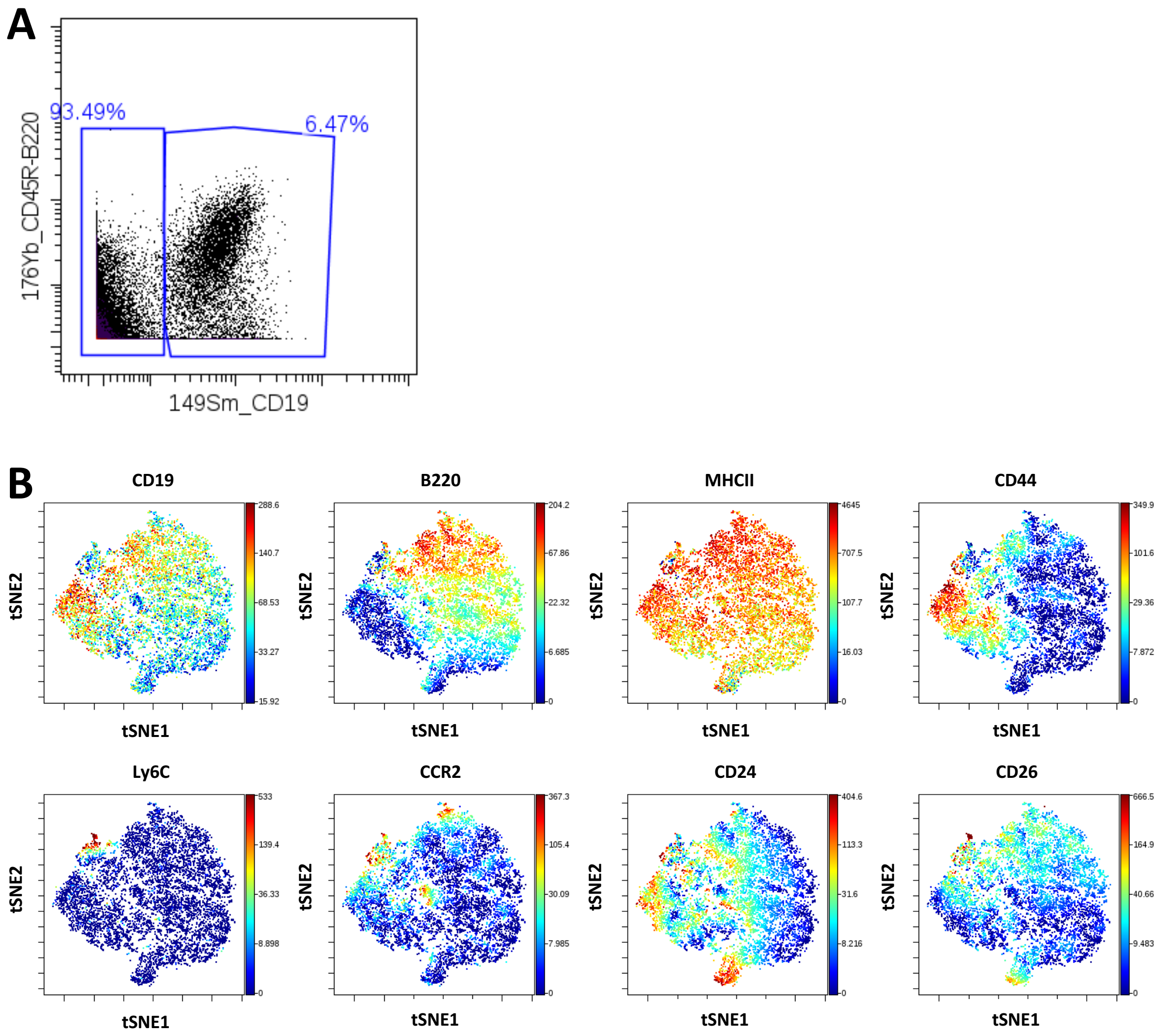

Figure S4 B cells exhibit different expression profiles of key markers. A. Dot plot showing B cells gating ($CD19^{+}B220^{-/+}$ from live Lin-CD45+ leucocytes). **B.** B cells were clustered using viSNE on the expression of 10 cell surface and intracellular markers outlined in supplemental table 1. Expression levels of selected myeloid markers in the resulting viSNE clustered cell populations is shown.

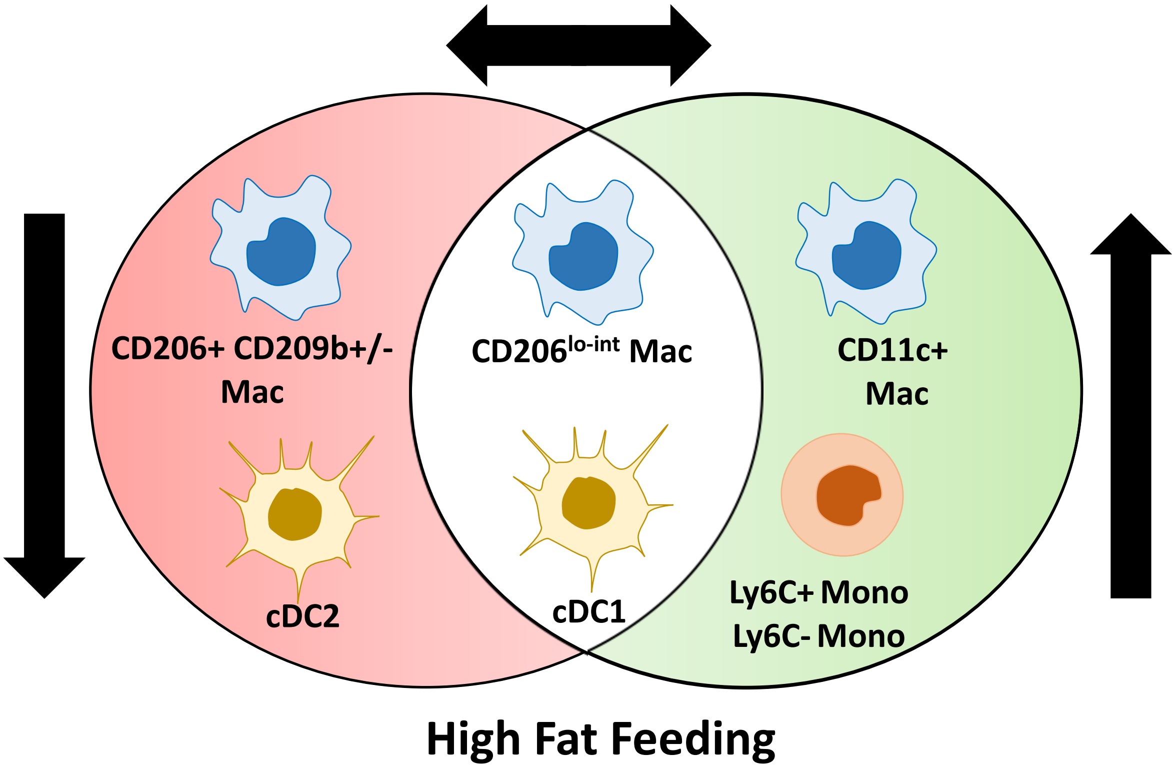

Figure S5 Schematic diagram showing how myeloid cell populations in atherosclerotic aortas of ApoE-/- mice are affected by high fat feeding. High fat feeding of ApoE-/- mice induces changes in the proportion of several myeloid cell subsets in the aorta as identified by viSNE analysis. $Ly6C^{+}$ and $Ly6C^{-}$ monocytes as well as $CD11c^{+}$ macrophages were increased in aortas of high fat fed mice (green circle) and conventional type 2 dendritic cells (cDC2) and $CD206^{+}CD169^{+}CD209^{+/-}$ macrophages were reduced (red circle). Proportions of conventional type 1 dendritic cells (cDC1), and $CD206^{lo\text{-}int}$ subsets were unchanged by high fat feeding.
